# A prospective study of surgeons’ workloads and associated factors in real-world practice

**DOI:** 10.1038/s41598-024-59596-1

**Published:** 2024-04-28

**Authors:** Shigeru Harada, Takashige Abe, Jun Furumido, Keita Takahashi, Kanta Hori, Noriyuki Abe, Masafumi Kon, Sachiyo Murai, Haruka Miyata, Hiroshi Kikuchi, Ryuji Matsumoto, Takahiro Osawa, Nobuo Shinohara

**Affiliations:** 1 Department of Urology, Hokkaido University Graduate School of Medicine, North-15, West-7, North Ward, Sapporo, 060-8638 Japan; 2 Department of Urology, Asahikawa Kousei Hospital, Asahikawa, Japan; 3 Department of Biostatistics, Hokkaido University Graduate School of Medicine, Sapporo, Japan; 4 Department of Urology, Asahikawa Medical University, Asahikawa, Japan

**Keywords:** Health care, Health occupations, Medical research, Urology

## Abstract

New technologies such as laparoscopic and robotic surgery are spreading, and there is a demand for physicians to keep up with novel methods. In contrast to the recent focus on healthcare professional burnout, the mental and physical costs during surgery are not well-understood. We aimed to quantify surgeons’ workloads in daily urological surgical practice and clarify potential background factors associated with such workloads. Urologists in Hokkaido, Japan, were invited to this study. Between December 2020 and December 2021, participants repeatedly reported workloads, which were assessed using the National Aeronautics and Space Administration-Task Load Index (NASA-TLX), after each surgery in conjunction with participants’ names, patients’ backgrounds, their roles (independent operator, operator under supervision, instructor, and 1st or 2nd assistant), and surgical outcomes, via SurveyMonkey^®^. Because of the heterogeneity among individuals, a linear mixed-effects model was utilized to analyze factors associated with NASA-TLX, calculating the parameter estimates (PE) of regression coefficients for each factor and their 95% confidence interval (CI). Sixty-five urologists (5 women) joined the study, and 2169 data were collected within 7 days after surgeries. A linear mixed-effects model revealed that female surgeons (PE + 15.56, 95% CI 2.36–28.77), urgent/emergency surgery (PE + 6.65, 95% CI 4.59–8.70), intraoperative complications (PE + 9.26, 95% CI 6.76–11.76), and near-miss incidents (PE + 3.81, 95% CI 2.27–5.36) were associated with higher workloads. Regarding the surgeons’ role, operator under supervision (PE + 12.46, 95% CI 9.86–15.06) showed the highest workloads. Surgeons’ workloads decreased as the number of previous cases of the same procedure increased. Surgeons’ workloads were associated with various factors. Given that the highest workloads were for operators under supervision, instructors should be aware of trainees’ high workloads and devise appropriate instructional interventions.

## Introduction

Recently, healthcare professional burnout has been emphasized^1–8^. For example, Pulcrano M et al.^3^ reported in their systematic review across multiple surgical specialties that high emotional exhaustion was observed in up to 31% of attending surgeons versus up to 42% of residents, high depersonalization was observed in up to 26% of attending surgeons versus up to 53% of residents, and a low personal sense of accomplishment was observed in up to 54% of attending surgeons versus up to 55% of residents. Al-Ghunaim et al.^4^ reported in their meta-analysis that surgeon burnout was significantly correlated with a higher incidence of medical errors. Symptoms of burnout were also noted in urology^1,2,5,6^. Harris AM et al.^2^ observed that, based on the American Urological Association census survey, in 2021, 36.7% of urologists reported burnout according to the Maslach Burnout Inventory, compared with 36.2% in 2016.

In surgical care including urology, according to the spreading of new technologies such as laparoscopic and robotic surgery, there is a demand for physicians to keep up with these emerging novel methods, and the mental and physical costs during surgery have also drawn scientific interest. Law et al. recently reported that a robot-assisted approach was associated with less mental demand, physical demand, and effort than open or laparoscopic after assessing the National Aeronautics and Space Administration Task Load Index (NASA-TLX) to self-assess workload following abdominopelvic colon and rectal surgeries^9^.

Beyond question, the operating room is a place where marked workloads are placed on medical staff, and surgeons frequently have to address difficult problems under time pressure. In order to promote the well-being of surgeons, develop better training curriculums, and improve surgical performance and patient safety, we consider that it is paramount to better understand surgeons’ mental workloads (MWLs). In the present study, we quantified surgeons’ workloads in daily urological surgical practice, and clarified potential background factors associated with MWLs.

## Methods

This prospective study was approved by the Institutional Review Board at Hokkaido University Hospital Clinical Research Administration Center (No. 020-0159) and conducted in accordance with the Declaration of Helsinki as well as the Ethical Guidelines for Medical and Health Research Involving Human Subjects. Urologists practicing at Hokkaido University Hospital and affiliated institutions were recruited for the present study by email. After obtaining written informed consent regarding the use of their data for research, participants’ demographic data were collected via an online survey tool (SurveyMonkey^®^, https://www.surveymonkey.com/), such as: age, sex, year of graduation from medical university, years of clinical experience in urology, board-certified urologist, certification of attending doctor, that of laparoscopic surgery, and that of robotic surgery. Previous surgical experiences as a primary surgeon or an instructor (leading role) were also collected regarding transurethral, open, laparoscopic, or robotic surgery.

### NASA-TLX collection

Between December 2020 and December 2021, after each surgical case, participants reported their NASA-TLX scores via SurveyMonkey^®^, using the original version in English. NASA-TLX includes six subscales: mental, physical, and temporal task demands, as well as effort, frustration, and perceived performance, and the questionnaire uses a 20-point visual analogue scale to measure MWLs based on the 6 abovementioned subscales^10^. Participants’ names, operative procedure, previous surgical experience of the same procedure as a primary or leading surgeon, surgical role (independent operator, operator under supervision, instructor, and 1st or 2nd assistant), patients’ backgrounds (age, sex, and comorbidities), surgical information (date, elective or urgent/emergency surgery, operative time, blood loss, and order of surgery on the day), intraoperative complications, near-miss incidents (such as anatomical misidentification, surgical equipment malfunction, trouble with anesthesia, intraoperative telephone calls, communication errors in the surgical team, etc.), subjective procedure difficulty, and on-call duty last night, were also collected. Furthermore, participants were allowed to describe additional surgical findings in the free-description column, such as: adhesive visceral fat due to obesity, difficulty due to patients’ body size, marked adhesion in the abdominal cavity, and collaborative surgery with other disciplines, and such information was classified as “other difficulty” for subsequent analysis. In this study, because we collected NASA-TLX scores not only from operators, but also from assistants, there could be overlapping patients in the present dataset. During the study period, one of the authors (SH) emailed the total number of reported cases at the end of every month to participants as a study reminder.

### Data analysis

During the 13 months of the study period, NASA-TLXs were collected from the participants after surgeries. Considering the effect of time-dependent memory loss, we included data reported within 7 days after surgery in the present analysis. Cases with missing data were excluded from the analysis.

In the present study, we aimed to explore the overall relationship between mental workloads of urologic surgeons and potentially influencing factors abovementioned. Because of the heterogeneity among individuals, we utilized a linear mixed-effects model, a statistical model containing both fixed and random effects, to analyze the factors associated with NASA-TLX, estimating the regression coefficients for each factor and calculating their 95% confidence interval^11^. We included an individual participant identifier as a random effect, considering the subjective tendency of reporting high or low NASA-TLX scores. We then included surgeons’ sex, previous experience (years), information on certificates (laparoscopic or robotic surgery), previous surgical experiences of the same procedure, surgical role (independent operator, operator under supervision, instructor, first assistant, or second assistant), order of surgery on the day, on-call duty last night, patients’ information (age, sex, history of hypertension, diabetes, cardiovascular disease, cerebrovascular disease, cancer, and previous surgical experience), surgical situation (elective or emergency surgery), surgical approach (open, laparoscopic, robotic, transurethral, or others), surgical outcomes (operative time, blood loss, intraoperative complications, and near-miss incidents), and other difficulties as covariates with fixed effects. For combination surgeries involving multiple procedures performed simultaneously, two of the authors (SH and TA) discussed and classified them. For example, in combination surgeries like robot-assisted radical cystectomy and laparoscopic radical nephroureterectomy, we classified it as “Robotic”. All calculations were performed using JMP^®^ Pro, version 17.0.0 (SAS Institute, Cary, NC, USA). Descriptive statistics were also calculated using JMP Pro, version 17.0.0. *P* < 0.05 was considered significant.

## Results

During the study period, a total of 2372 surgeries were reported from 67 participants. Of those, 2185 (92.1%) were reported within 7 days after surgery by 65 urologists [1384 (58.4%) were reported on the day of surgery, 317 (15.6%) were on the next day, and 430 (18.1%) were 2–7 days later]. Of the 2185 cases, after excluding 16 cases with missing data, 2169 cases (91.4%) were finally included in the analysis. The median number of reports per surgeon was 17 (range, 1–177). Ninety-five cases (4.4%) were reported by 5 female surgeons. Table 1 summarizes the backgrounds of 65 participants. The median age was 42 years (range, 27–61), and the median years of previous clinical experience was 15 (range, 1–36). Female surgeons accounted for 7.7% (5/65). Regarding skill qualification, 28 (43.1%) had the endoscopic surgical skill qualification authorized by Japanese Society of Endourology and Robotics^12^, and 40 (61.5%) had Da Vinci^®^ certification.

**Table 1:** Summary of the 65 participants’ backgrounds.

Characteristics	Results
Age, years, median (range)	42 (27–61)
Sex	
Male, n (%)	60 (92.3)
Female, n (%)	5 (7.7)
Surgeons’ experience, years, median (range)	15 (1–36)
Board-certified urologist, n (%)	46 (70.8)
Board-certified instructor, n (%)	42 (64.6)
Endoscopic surgical skill qualification, n (%)	28 (43.1)
Da Vinci certificate, n (%)	40 (61.5)

Female surgeons accounted for 7.7% (5/65). The median age was 42 years (range, 27–61), and the median years of previous clinical experience was 15 (range, 1–36). Surgeons at a variety of career levels, including trainees, participated in this study.

Table 2 summarizes the 2169 surgeries collected in this study. Elective surgery accounted for 90.3% (1959/2169), and emergency surgery for 9.7% (210/2169). Regarding the surgical approach, “Open” accounted for 11.9% (259/2169), “Laparoscopic” for 14.8% (321/2169), “Robotic” for 19.1% (415/2169), “Transurethral” for 33.8% (733/2169), and other procedures for 20.3% (441/2169). Regarding the surgeons’ role, operator (independent) accounted for 23.1% (501/2169), operator under supervision for 28.8% (625/2169), 1st assistant as an instructor for 30.4% (659/2169), 1st assistant for 10.5% (228/2169), and 2nd assistant for 7.2% (156/2169). Surgeons reported subjective procedural difficulty levels as follows: 64.7% as expected (1403/2169), 25.0% more difficult than expected (542/2169), and 10.2% less difficult than expected (221/2169). Intraoperative complications were reported in 6.4% (138/2169), including injuries of other organs (n = 61), urethral injuries during transurethral surgeries (n = 34), massive bleeding (n = 19), vascular problems (n = 14), and others (n = 10). Regarding near-miss incidents, intraoperative telephone calls were the most frequent (49.4%, 310/627), followed by malfunctions and connection problems with surgical forceps and devices (19.6%, 123/627), and anatomical misidentifications (8.1%, 51/627) (Supplementary Table 1). In terms of other difficulties, marked adhesion in the abdominal cavity was the most common at 35.4% (69/195), followed by experiencing any difficulty or exhaustion during the procedure at 24.1% (47/195), and surgery with an unscheduled procedure at 11.3% (22/195) (Supplementary Table 2). Supplementary Tables 3 and 4 summarize patients’ histories other than previous surgery and cancer, and details of surgical procedures.

**Table 2:** Summary of the 2169 surgical cases collected.

Characteristics	n(%)
Patient age, years, median (range)	71 (0–100)
Patient sex	
Male	1595 (73.5%)
Female	574 (26.5%)
Patient history	
Hypertension	
Yes	1019 (47.0%)
No	1150 (53.0%)
Diabetes	
Yes	397 (18.3%)
No	1772 (81.7%)
Cardiovascular disease	
Yes	383 (17.7%)
No	1786 (82.3%)
Cerebrovascular disease	
Yes	222 (10.2%)
No	1947 (89.8%)
Previous surgery	
Yes	565 (26.0%)
No	1604 (74.0%)
Cancer	
Yes	361 (16.6%)
No	1808 (83.4%)
Type of surgery	
Urgent or emergency surgery	210 (9.7%)
Elective surgery	1959 (90.3%)
Order of surgery on the day	
1st	1425 (65.7%)
2nd	547 (25.2%)
3rd or after	197 (9.1%)
On-call duty last night	
Yes	128 (5.9%)
No	2041 (94.1%)
Surgical approach	
Open	259 (11.9%)
Laparoscopic	321 (14.8%)
Robotic	415 (19.1%)
Transurethral	733 (33.8%)
Others	441 (20.3%)
Previous surgical experiences of the same procedure	
0	106 (4.9%)
1–10	337 (15.5%)
11–50	561 (25.9%)
51–100	279 (12.9%)
101–500	613 (28.3%)
501 or more	273 (12.6%)
Role of surgeon	
Independent operator	501 (23.1%)
Operator under supervision	625 (28.8%)
Instructor	659 (30.4%)
1st assistant	228 (10.5%)
2nd assistant	156 (7.2%)
Surgical outcomes	
Operative time, min, median (range)	110 (1–853)
Blood loss, mL, median (range)	0 (0–16,000)
Intraoperative complication	
Yes	138 (6.4%)
No	2031 (93.6%)
Near-miss incident	
Yes	482 (22.2%)
No	1687 (77.8%)
Other difficulty	
Yes	186 (8.6%)
No	1983 (91.4%)
Subjective surgical difficulty	
More difficult	542 (25.0%)
Less difficult	221 (10.2%)
As difficult	1403 (64.7%)
No answer	3 (0.1%)

Urgent or emergency surgery accounted for 9.7% (210/2169). Regarding the condition of the surgeon, On-call duty last night accounted for 5.9% (128/2169). Concerning previous surgical experience of the same procedure, in 106 cases (4.9%), surgeons participated in the procedure for the first time in their careers. In terms of the surgical approach, “Open” accounted for 11.9% (259/2169), “Laparoscopic” for 14.8% (321/2169), “Robotic” for 19.1% (415/2169), “Transurethral” for 33.8% (733/2169), and other procedures for 20.3% (441/2169). Regarding the role in surgery, surgeons reported cases in which they participated not only as operators but also as assistants. Details of intraoperative complications were as follows: injuries of other organs = 61, urethral injuries during transurethral surgeries = 34, massive bleeding = 19, vascular problems = 14, and others = 10.

### Factors associated with NASA-TLX scores

**Figure 1:**
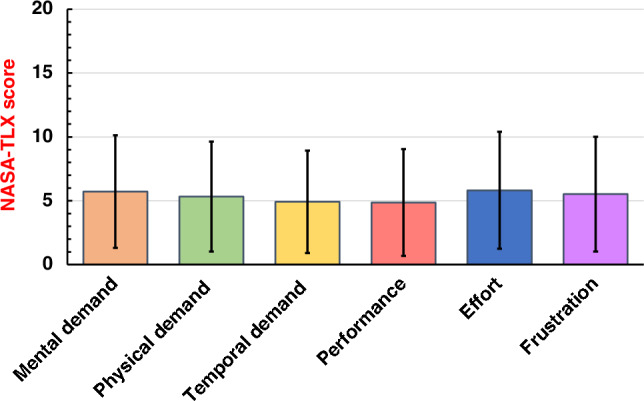
Comparison of mean NASA-TLX subscales Effort was the highest reported subscale (mean 5.82, standard deviation [SD] 4.58), followed by mental demand (mean 5.72, SD 4.41), and frustration (mean 5.52, SD 4.49). Error bars in the graph represent the standard deviation.

**Figure 2:**
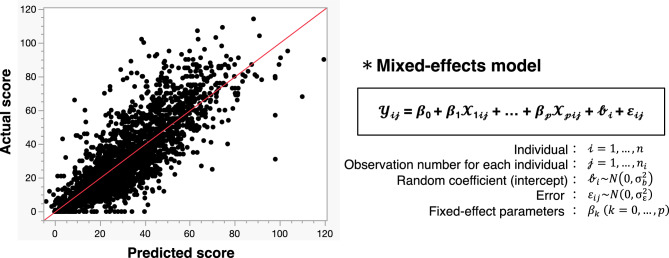
Scatter plots between actual and predicted NASA-TLX scores based on the present linear mixed-effects model.

**Figure 3:**
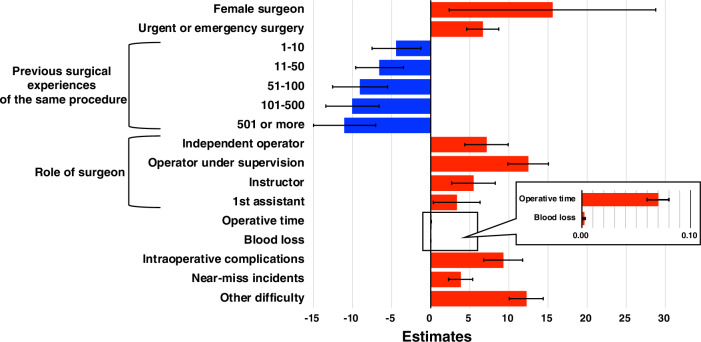
Waterfall-plot of the significant estimates (slopes) of the present mixed-effects model Red bars represent the impact on higher workloads, and blue bars represent the impact on lower workloads. Error bars in the graph represent 95% confidence intervals. Operative time and blood loss are shown enlarged because the length of each bar is very short. Female surgeons, urgent or emergency surgery, and unexpected intraoperative events (intraoperative complications and near-miss incidents) were associated with higher workloads. In terms of the surgeon’s role, operator under supervision had the highest workload. As previous surgical experiences of the same procedure increased, surgeons’ workloads decreased.

Overall, the 65 participants reported a mean NASA-TLX score of 32.2 (standard deviation [SD]: 22.7) for 2169 surgeries in total. Figure 1 shows a comparison of mean NASA-TLX subscales. Effort was the highest reported subscale (mean: 5.82, SD 4.58), followed by mental demand (mean 5.72, SD 4.41), and frustration (mean: 5.52, SD 4.49). To identify factors associated with NASA-TLX scores, we utilized a linear mixed-effects model. Figure 2 shows scatter plots between actual and predicted NASA-TLX scores based on the present linear mixed-effects model. The random coefficients (intercepts) varied widely among the participants (Supplementary Fig. 1). Table 3 summarizes the estimates (slopes) of fixed effects, and Fig. 3 shows a waterfall-plot of the significant characteristics. Female surgeons showed higher NASA-TLX scores (Parameter estimate [PE] + 15.56, 95% confidence interval [CI] 2.36–28.77; *p* = 0.022) than male surgeons. While urgent/emergency surgery was associated with higher MWLs (PE + 6.65, 95% CI 4.59–8.70; *p* < 0.001) compared with elective surgery, the order of surgery on the day showed no significant correlation with MWLs. MWLs decreased with increased previous surgical experiences of the same procedure, although years of previous surgical experience did not show a significant correlation with MWLs. Regarding the surgeons’ role (reference standard: 2nd assistant), operator under supervision showed the highest MWLs (PE + 12.46, 95% CI 9.86–15.06; *p* < 0.001), followed by independent operator (PE + 7.12, 95% CI 4.34–9.91; *p* < 0.001), 1st assistant as an instructor (PE + 5.46, 95% CI 2.67–8.25; *p* < 0.001), and 1st assistant (PE + 3.32, 95% CI 0.33–6.31; *p* = 0.030). Furthermore, NASA-TLX scores were increased by + 0.07 per minute of operative time (95% CI 0.06–0.08; *p* < 0.001), and + 0.002 per mL of blood loss (95% CI 0.001–0.003; *p* < 0.001), and higher for unexpected intraoperative events [+ 9.26 higher for intraoperative complications (95% CI 6.76–11.76; *p* < 0.001), and + 3.81 higher for near-miss incidents (95% CI 2.27–5.36; *p* < 0.001)]. Other difficulties were also associated with higher MWLs (PE + 12.22, 95% CI 10.04–14.39; *p* < 0.001). Surgeons’ experience, technical qualifications, on-call duty, and patients’ factors (age, sex, history) did not show significant correlations with MWLs.

**Table 3:** Summary of estimates of the present multiple linear mixed-effects model.

Fixed effect	n(%)	Estimate (95% CI)	*p* value
Surgeon sex			
Male	60 (92.3%)	Reference	–
Female	5 (7.7%)	+ 15.56 (2.36–28.77)	0.022
Surgeon experience, years, median (range)	15 (1–36)	− 0.25 (− 0.62–0.12)	0.184
Endoscopic surgical skill qualification			
Yes	28 (43.1%)	+ 3.61 (− 4.20–11.43)	0.358
No	37 (56.9%)	Reference	–
Da Vinci certificate			
Yes	40 (61.5%)	− 2.06 (− 9.00–4.89)	0.556
No	25 (38.5%)	reference	–
Patient age, years, median (range)	71 (0–100)	− 0.02 (− 0.06–0.01)	0.225
Patient sex			
Male	1595 (73.5%)	Reference	–
Female	574 (26.5%)	+ 0.68 (− 0.68–2.03)	0.329
Patient history			
Hypertension			
Yes	1019 (47.0%)	+ 0.97 (− 0.30–2.24)	0.133
No	1150 (53.0%)	Reference	–
Diabetes			
Yes	397 (18.3%)	+ 0.55 (− 0.98–2.08)	0.4823
No	1772 (81.7%)	Reference	–
Cardiovascular disease			
Yes	383 (17.7%)	+ 1.04 (− 0.53–2.61)	0.193
No	1786 (82.3%)	Reference	–
Cerebrovascular disease			
Yes	222 (10.2%)	− 0.16 (− 2.11–1.79)	0.8742
No	1947 (89.8%)	Reference	–
Previous surgery			
Yes	565 (26.0%)	− 0.89 (− 2.30–0.52)	0.215
No	1604 (74.0%)	Reference	–
Cancer			
Yes	361 (16.6%)	− 0.07 (− 1.71–1.58)	0.937
No	1808 (83.4%)	Reference	–
Type of surgery			
Urgent or emergency surgery	210 (9.7%)	+ 6.65 (4.59–8.70)	< 0.001
Elective surgery	1959 (90.3%)	Reference	–
Order of surgery on the day			
1st	1425 (65.7%)	Reference	–
2nd	547 (25.2%)	− 0.29 (− 1.65–1.07)	0.676
3rd or after	197 (9.1%)	+ 1.00 (− 1.14–3.14)	0.359
On-call duty last night			
Yes	128 (5.9%)	− 0.95 (− 3.46–1.55)	0.455
No	2041 (94.1%)	Reference	–
Surgical approach			
Open	259 (11.9%)	− 0.41 (− 3.22–2.41)	0.778
Laparoscopic	321 (14.8%)	+ 2.33 (− 0.09–4.76)	0.059
Robotic	415 (19.1%)	+ 2.62 (− 0.001–5.23)	0.050
Transurethral	733 (33.8%)	Reference	–
Others	441 (20.3%)	− 0.29 (− 2.10–1.52)	0.754
Previous surgical experiences of the same procedure			
0	106 (4.9%)	Reference	–
1–10	337 (15.5%)	− 4.39 (− 7.52–− 1.27)	0.006
11–50	561 (25.9%)	− 6.55 (− 9.61–− 3.50)	< 0.001
51–100	279 (12.9%)	− 9.04 (− 12.55–− 5.53)	< 0.001
101–500	613 (28.3%)	− 10.02 (− 13.42–− 6.61)	< 0.001
501 or more	273 (12.6%)	− 11.03 (− 15.01–− 7.05)	< 0.001
Role of surgeon			
Independent operator	501 (23.1%)	+ 7.12 (4.34–9.91)	< 0.001
Operator under supervision	625 (28.8%)	+ 12.46 (9.86–15.06)	< 0.001
Instructor	659 (30.4%)	+ 5.46 (2.67–8.25)	< 0.001
1st assistant	228 (10.5%)	+ 3.32 (0.33–6.31)	0.030
2nd assistant	156 (7.2%)	Reference	–
Surgical outcomes			
Operative time, min, median (range)	110 (1–853)	+ 0.07 (0.06–0.08)	< 0.001
Blood loss, mL, median (range)	0 (0–16,000)	+ 0.002 (0.001–0.003)	< 0.001
Intraoperative complication			
Yes	138 (6.4%)	+ 9.26 (6.76–11.76)	< 0.001
No	2031 (93.6%)	Reference	–
Near-miss incident			
Yes	482 (22.2%)	+ 3.81 (2.27–5.36)	< 0.001
No	1687 (77.8%)	Reference	–
Other difficulty			
Yes	186 (8.6%)	+ 12.22 (10.04–14.39)	< 0.001
No	1983 (91.4%)	Reference	–

A mixed effects model was conducted to identify factors associated with NASA-TLX scores, and the parameter estimates (PE) and 95% confidence intervals (CI) for each fixed effect are presented. *P* < 0.05 was considered significant, shown in bold.

## Discussion

Recently, burnout among surgeons has been well-recognized and reported to be influenced by multifactorial backgrounds. Shanafelt TD et al. reported that, in an anonymous survey involving members of the American College of Surgeons with a response rate of 32% (7905/24,922), a high rate of burnout syndrome (severe emotional exhaustion and depersonalization) was observed in 40% of respondents, and a younger age, having children, the area of specialization, number of nights on call per week, hours worked per week, and having compensation determined entirely based on billing were independent factors^7^. Chati R et al. observed that, in an anonymous questionnaire survey involving French digestive surgeons with a response rate of 65.6% (328/500), 62% of the surgeons had burnout syndrome, and a multivariate model revealed that perceived aggression from patients, lack of gratitude on the part of seniors, a level of responsibility deemed unsuitable, and the absence of extracurricular activities were independent adverse factors^8^.

Operations demand marked workloads and surgeons frequently have to tackle with difficult situations under time pressure. As abovementioned, in order to better understand surgeons’ MWLs in daily clinical practice, we prospectively collected surgeons’ MWLs in real-world surgical cases with a wide range of procedure types, which were performed by urologic surgeons at a variety of career levels, including trainees. As data were repeatedly collected from each participant (not only from operators, but also from assistant surgeons) via an online survey tool with a registered form (with individual names), we utilized a linear mixed-effects model, which included an individual participant identifier as a random effect, and evaluated factors (fixed effects) related to subjective MWLs assessed by NASA-TLX, considering individual tendencies at the time of reporting MWLs. As expected, the random effects varied widely among the present participants (range: − 20.94 to + 24.19); in other words, each participant had a certain tendency of reporting high or low NASA-TLX scores.

Regarding the surgeons’ role, operators showed higher MWLs compared with the reference standard of the 2nd assistant. Furthermore, operators under supervision showed the highest MWLs. Our observations may reflect two aspects: one is that operators perform difficult cases, and they need senior doctors’ advice; and the other is that operators are in a training phase, and they still need continuous supervision. In both situations, in order to optimize surgical outcomes and education, supervising instructors should understand trainees’ high MWLs under their supervision, and prevent excessive workloads by adopting an appropriate attitude.

In this study, female surgeons showed higher MWLs than male surgeons. While the present cohort included a limited number of female surgeons (n = 5, 95 workload data), we have certain concerns regarding the current observations. Sex inequality in the workplace remains an unresolved problem, including surgeons and surgical trainees^13^. Joh DB et al. analyzed 119,380 general surgery procedures performed by 120 trainees (male = 78, female = 42) in New Zealand between 2012 and 2017. Operative autonomy was classified by the trainees for each procedure according to 5 levels: (1) Assisting senior resident, (2) Assisting surgeon mentor, (3) Primary operator with surgeon mentor scrubbed, (4) Primary operator with surgeon mentor in the operating room, (5) Primary operator with surgeon mentor available. When defining meaningful autonomy as levels 4 and 5, female trainees performed fewer procedures with meaningful autonomy than male trainees^14^. Meyerson et al. utilized a smartphone app and collected operative autonomy assessments according to the 4-point Zwisch scale (1. Show and tell, 2. Active help, 3. Passive help, 4. Supervision only). During the 9-month study period, 412 residents and 524 faculty members evaluated 8900 cases, and female residents received less autonomy from faculty members than male residents overall^15^. Furthermore, in terms of professional burnout in urology, female urologists reported a higher prevalence of burnout^2,6^. At present, although we do not have a clear explanation for higher intraoperative MWLs in our female cohort, we believe that autonomy in the operating room is a pivotal part of surgical training, which helps trainees develop confidence and realize their current limitations, and mentors should determine the degree of trainees’ latitude based on their current competency, not sex bias. Gas et al.^6^ reported very important findings based on self-assessment questionnaires sent to trainees of the French Association of Urologists, whereby a “Feeling of being well-trained” was the only protective factor against emotional exhaustion, depersonalization, and negative feelings of personal accomplishment.

Regarding the length of surgery, NASA-TLX scores increased by + 0.07 per minute of surgical time. Rieger et al.^16^ also reported a similar observation. They evaluated surgeons’ intraoperative workloads through a mobile health system, such as heart rate, breathing rate, and skin temperature, and NASA-TLX scores. Ten surgeons participated in their 24-h monitoring study, and the length of surgery had a significant impact on NASA-TLX scores, while working as primary surgeons did not result in higher workloads. Although intermittent breaks during surgery are not common, several researchers reported promising findings^17,18^. Engelmann C et al., in their prospective randomized study, assigned 51 operations randomly to an investigational group with intraoperative breaks or a conventional group without intraoperative breaks, and the investigational group showed lower stress hormone levels, intraoperative events, and error-performance scores, without prolonging the operative duration^18^.

In this study, near-miss incidents were associated with higher MWLs. In Supplementary Table 1, these events mostly represented distractions during surgeries. According to the cognitive load theory, a well-known learning theory proposed by Sweller et al., the present near-miss incidents could be categorized into *Extraneous loads*. Our observation, in which NASA-TLX scores decreased according to increased previous caseloads of the same procedure, should reflect the decrease of *Intrinsic load* which comes from difficulty, complexity, or prior experience of the procedure. To maximize learning chances of trainees, in other words, maximize *Germane load* for learning*,* we consider that extraneous loads should be minimized during surgery. Unnecessary intraoperative disruptions such as telephone calls should be avoided as much as possible.

This study had several limitations. First, because data collection was performed in a voluntary manner, we could not collect workload data from all surgical cases during the study period. Thus, there may be a bias between reported and non-reported cases. Second, we included only 5 female participants, and the wide range of CI (2.36–28.77) may be related to this small number. We need to assess a larger cohort in the future to confirm our findings. Third, this study was performed among Japanese urologists in Hokkaido Prefecture, and so our observations may be biased due to this cohort. Similar studies are warranted to assess whether the present findings are universal. Fourth, during the study period, we had the novel coronavirus disease (COVID) 2019 pandemic. Although Japan did not experience lockdown measures and mortality due to COVID-19 infection remined low (0.2%), surgeons’ workloads may have been indirectly influenced. Fifth, in addition to surgery, urologists have numerous other duties such as outpatient clinics, in-hospital patient management, on-call/night-shifts, academic jobs, and other miscellaneous work. Nevertheless, we believe that our observations facilitate a better understanding of surgeons’ MWLs in daily clinical practice, being helpful for promoting the well-being of surgeons, developing better training curriculums, and improving surgical performance and patient safety.

## Conclusion

Surgeons’ MWLs were associated with various factors. Regarding the surgeons’ role, operators showed higher MWLs, especially under supervision. Instructors should be aware of the high MWLs of trainees under their supervision and devise appropriate instructional interventions.

### Supplementary Information

Supplementary Information.

## Data Availability

The datasets generated and/or analyzed during the current study are not publicly accessible, but are available from the corresponding author on reasonable request.

## Abbreviations

CI: Confidence interval
COVID-19: Coronavirus disease 2019
MWLs: Mental workloads
NASA-TLX: National aeronautics and space administration-task load index
PE: Parameter estimates
SD: Standard deviation
