# Supplementary material for: A prospective study of surgeons’ workloads and associated factors in real-world practice
Source: Sci Rep. 2024 Apr 28;14:9741. doi: 10.1038/s41598-024-59596-1 (PMC11056359; doi:10.1038/s41598-024-59596-1)
Supplement: Supplementary file 1 — Supplementary Information. [file 41598_2024_59596_MOESM1_ESM.pdf]

**Supplementary Table 1. Summary of near-miss incidents**

| Incidents | n | % |
|---|---|---|
| Intraoperative answering of a telephone call | 310 | 49.4 |
| Malfunctions and connection problems with surgical forceps and devices | 123 | 19.6 |
| Anatomical misidentification | 51 | 8.1 |
| Unexpected heavy bleeding | 50 | 8.0 |
| Communication problems among surgeons and surgical team | 35 | 5.6 |
| Sponge/Needle count discrepancy | 23 | 3.7 |
| Anesthesia-related problems | 21 | 3.3 |
| Sudden deterioration of patient's condition | 9 | 1.4 |
| Temporary loss of needles or instruments | 4 | 0.6 |
| Port site problems | 1 | 0.2 |

* Several near-miss incidents simultaneously reported in one case.

**Supplementary Table 2. Summary of other difficulties**

| Difficulties | n | % |
|---|---|---|
| Marked adhesion in the abdominal cavity | 69 | 35.4 |
| Experiencing any difficulty or exhaustion during the procedure | 47 | 24.1 |
| Surgery with an unscheduled procedure | 22 | 11.3 |
| Difficulty due to patients' body size | 16 | 8.2 |
| Collaborative surgery with other disciplines | 13 | 6.7 |
| Redo surgery | 9 | 4.6 |
| Adhesive visceral fat | 7 | 3.6 |
| Poor physical condition of the surgeon | 5 | 2.6 |
| Conversion to open surgery | 4 | 2.1 |
| Anatomical abnormalities | 3 | 1.5 |

* Several other difficulties simultaneously reported in one case.

**Supplementary Table 3. Patients' histories**

| **Comorbidities** | n |
|---|---|
| Hypertension | 1019 |
| Previous surgery | 565 |
| Diabetes | 397 |
| Cardiovascular disease | 383 |
| Cancer | 361 |
| Cerebrovascular disease | 222 |
| Chronic kidney disease | 85 |
| Non hemodialysis | 60 |
| Hemodialysis | 25 |
| Gastrointestinal disease (benign) | 65 |
| Orthopedic disease (benign) | 62 |
| Pulmonary respiratory disease (benign) | 50 |
| Dyslipidemia | 47 |
| Mental illness/Dementia | 47 |
| Urological disease (benign) | 33 |
| Ophthalmic disease | 29 |
| Hyperuricemia | 28 |
| Neurodegenerative disease | 23 |
| Metabolic endocrine disease | 21 |
| Obesity | 19 |
| Rheumatoid arthritis | 14 |
| Otorhinolaryngological disease (benign) | 11 |
| Infectious disease | 9 |
| Gynecological disease (benign) | 5 |
| Miscellaneous | 66 |
| None | 472 |

* Several comorbidities simultaneously reported in one case.

**Supplementary Table 4. Details of surgical procedures**

| Procedure | n |
|---|---|
| **Open (n=259)** | |
| **Cystectomy** | |
| Radical cystectomy (Ileal conduit) | 19* |
| Radical cystectomy (Cutaneous ureterostomy) | 15 |
| Radical cystectomy (Ileal neobladder) | 2 |
| Radical cystectomy (Cutaneous ureterostomy) + Nephroureterectomy (Open) | 5 |
| Radical cystectomy (Cutaneous ureterostomy) + Nephroureterectomy (Laparoscopic) | 3 |
| Radical cystectomy (without urinary diversion) + Nephroureterectomy (Open) | 1 |
| Partial cystectomy | 6† |
| **Prostatectomy** | |
| Radical prostatectomy | 68 |
| Suprapubic simple prostatectomy | 1 |
| **Radical nephroureterectomy** | 8 |
| **Nephrectomy** | |
| Radical nephrectomy | 25‡ |
| Simple nephrectomy | 3 |
| Partial nephrectomy | 12 |
| **Adrenalectomy** | 4 |
| **Resection of retroperitoneal tumors** | 7 |
| **Retroperitoneal lymph node dissection** | 6 |
| **Resection of urachal remnant** | 3 |
| **Transplantation surgery** | |
| Renal transplantation | 39 |
| Graftectomy | 3 |
| **Lithotomy** | |
| Cystolithotomy | 2 |
| Ureterolithotomy | 1 |
| **Pyeloplasty** | 1 |
| **Enterocystoplasty** | 2 |
| **Anastomosis** | |
| Ureterovesical | 4 |

| | |
|---|---|
| Ureter-ureter | 3 |
| Ureter-ileal conduit | 2 |
| **Suprapubic catheterization (Open cystotomy)** | 4 |
| **Vesicocutaneous fistula** | 2 |
| **Miscellaneous** | 8 |
| **Laparoscopic (n=321)** | |
| **Cystectomy** | |
| Radical cystectomy (Ileal conduit) | 1 |
| Radical cystectomy (Cutaneous ureterostomy) | 2 |
| **Radical nephroureterectomy** | 88 |
| **Nephrectomy** | |
| Radical nephrectomy | 84 |
| Simple nephrectomy | 5 |
| Partial nephrectomy | 22 |
| **Adrenalectomy** | |
| Adrenalectomy | 40 |
| Partial adrenalectomy | 1 |
| **Resection of retroperitoneal tumors** | 6 |
| **Retroperitoneal lymph node dissection** | 3 |
| **Resection of urachal remnant** | 7 |
| **Transplantation surgery** | |
| Donor nephrectomy | 47 |
| **Lithotomy** | |
| Ureterolithotomy | 2 |
| **Pyeloplasty** | 2 |
| **Varicocelectomy** | 2 |
| **Orchidopexy** | 1 |
| **Peritoneal dialysis catheter placement** | 4 |
| **Miscellaneous** | 4 |
| **Robotic (n=415)** | |
| **Cystectomy** | |
| Radical cystectomy (Ileal conduit) | 34§ |

| | |
|---|---|
| Radical cystectomy (Cutaneous ureterostomy) | 4 |
| Radical cystectomy (Ileal neobladder) | 4 |
| Radical cystectomy (without urinary diversion) | 3 |
| Radical cystectomy (Cutaneous ureterostomy) + Nephroureterectomy (Laparoscopic) | 5 |
| Radical cystectomy (Cutaneous ureterostomy) + Nephroureterectomy (Robotic) | 1 |
| **Prostatectomy** | |
| Radical prostatectomy | 247 |
| **Nephrectomy** | |
| Partial nephrectomy | 105 |
| **Sacrocolpopexy** | 7 |
| **Pyeloplasty** | 4 |
| **Anastomosis** | |
| Ureter-ureter | 1 |
| **Transurethral (n=733)** | |
| **Transurethral resection of bladder tumor (TURBT)** | |
| TURBT | 281 |
| TURBT + Upper urinary tract procedures[II] | 13 |
| **Transurethral resection of the prostate (TURP)** | |
| TURP | 60 |
| TURP + Transurethral cystolithotripsy | 3 |
| **Transurethral enucleation of the prostate with bipolar system (TUEB)** | 21 |
| **Transurethral lithotripsy** | |
| **Ureterolithotripsy** | 135 |
| **Cystolithotripsy** | 19 |
| **Transurethral electrocoagulation** | 11 |
| **Dilation** | |
| Ureteral dilation | 14 |
| Urethral dilation | 7 |
| **Transurethral incision** | |
| Bladder neck contracture | 4 |
| Urethral stricture | 1 |
| **Ureteroscopy** | 19 |
| **Bladder hydrodistention** | 8 |

| | |
|---|---|
| **Ureteral stent placement** | 127¶ |
| **Miscellaneous** | 10 |
| **Others (n=441)** | |
| **Vascular access surgery** | |
| Creation of vascular access (Arteriovenous fistula) | 68 |
| Permanent dialysis catheter placement | 2 |
| Thrombectomy of arteriovenous fistula | 2 |
| **Prostate needle biopsy** | |
| Transrectal prostate needle biopsy (TRPB) | 32 |
| Transperineal prostate needle biopsy (TPPB) | 11 |
| **Orchidopexy** | |
| Undescended testis | 45 |
| Testicular torsion | 15 |
| **Orchiectomy** | |
| Simple orchiectomy | 35 |
| Radical orchiectomy | 15 |
| **Hydrocelectomy** | 18 |
| **Spermatocelectomy** | 4 |
| **Varicocelectomy** | 1 |
| **Penectomy** | |
| Partial penectomy | 3 |
| Total penectomy | 2** |
| **Fiducial marker (Gold marker) placement** | 9 |
| **Surgery for phimosis** | |
| Circumcision | 9 |
| Dorsal slit of prepuce back | 5 |
| **Urethroplasty** | 7 |
| **Hypospadias repair** | 4 |
| **Artificial urinary sphincter insertion** | 5 |
| **Excision of caruncles** | 2 |
| **Tension-free vaginal tape (TVT) or Transobturator tape (TOT)** | 2 |
| **Tension-free vaginal mesh (TVM)** | 8 |
| **Vesicovaginal fistula repair** | 2 |

| | |
|---|---|
| **Percutaneous cystostomy** | 29 |
| **Percutaneous nephrostomy** | 37†† |
| **Percutaneous puncture of renal cyst** | 3 |
| **Percutaneous kidney biopsy** | 11‡‡ |
| **Peritoneal dialysis catheter placement/removal or creation of exit site** | 24 |
| **Central venous access port placement** | 7 |
| **Miscellaneous** | 24 |

* Contains one case of Total pelvic exenteration.

† Contains two cases requiring simultaneous ureterovesical anastomosis.

‡ Contains three cases requiring simultaneous thrombectomy.

§ Contains one case of Total pelvic exenteration.

|| Upper urinary tract procedures were as follows: ureteroscopy=7, ureteral stent placement=4, laser ablation of renal pelvis tumor=1, and retrograde pyelography=1.

¶ Contains one case of metallic stent placement.

** Contains one case requiring simultaneous sentinel lymph node biopsy.

†† Contains three cases requiring simultaneous ureteral stent placement, and one case of nephrostomy of transplanted kidney.

‡‡ Contains five cases of transplanted kidney biopsy.

TURBT, Transurethral resection of bladder tumor; TURP, Transurethral resection of the prostate; TUEB, Transurethral enucleation of the prostate with bipolar system; TRPB, Transrectal prostate needle biopsy; TPPB, Transperineal prostate needle biopsy; TVT, Tension-free vaginal tape; TOT, Transobturator tape; TVM, Tension-free vaginal mesh.

**Supplementary Figure 1. Box-plot of the random coefficients for years of surgeons' experience**

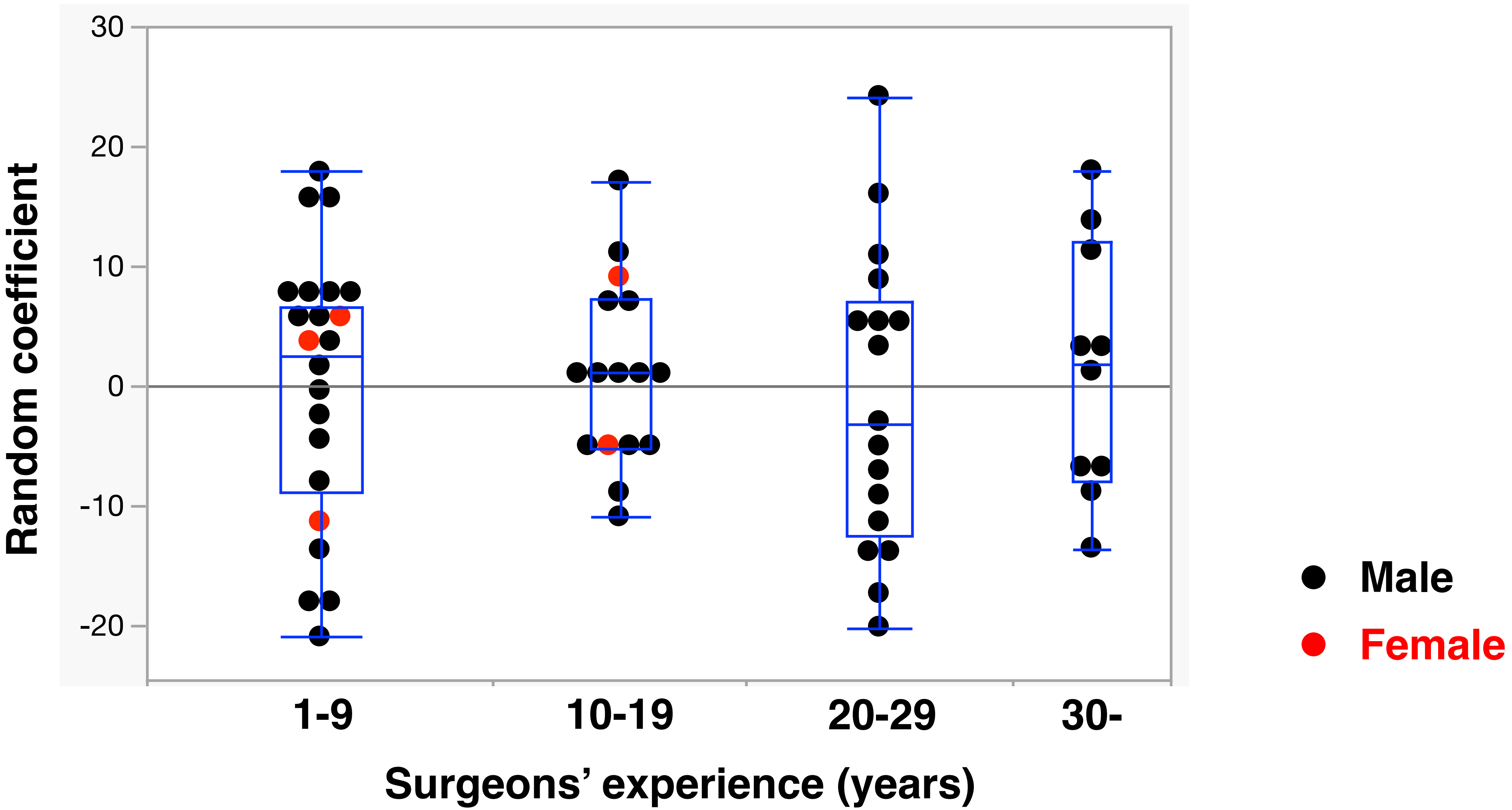

**Supplementary Figure 1. Box-plot of the random coefficients for years of surgeons' experience**

Black dots represent male surgeons, and red dots represent female surgeons. The random coefficients of the mixed-effects model did not differ among the 4 groups of years of surgeons' experience (1-9, 10-19, 20-29, and 30 or more years)
